# A COMMUNITY-BASED PARTICIPATORY APPROACH TO BUILDING ALLIANCE WITH LGBTQ2S+ OLDER ADULTS

**DOI:** 10.1093/geroni/igae098.1208

**Published:** 2024-12-31

**Authors:** Megan McCoy

**Affiliations:** Northern Arizona University, Northern Arizona University, Arizona, United States

## Abstract

The intersection of homophobia, transphobia, and heterosexism with ageism perpetuates health equity challenges for LGBTQ+ older adults. Many LGBTQ+ older adults fear having to “go back into the closet” to access healthcare, housing, and social support in later life and intentionally seek out identity-affirming settings. Yet, the availability of inclusive community spaces and services varies geographically. Understanding the experiences of LGBTQ+ older adults can inform research, advocacy, and programmatic efforts locally and regionally. Community-based participatory research (CBPR) is one framework for engaging historically silenced communities to drive research questions generated by and with the community. CBPR involves equitable community-academic partnerships, from development through dissemination. The first phase of successful CBPR involves forming a partnership and assessing community strengths, challenges, and dynamics— before we can even think about implementing a research project. This presentation will highlight how a CBPR model is used to engage key stakeholders to support LGBTQ2S+ older adults in Arizona better. Initially conceived as an advisory committee to inform research to build inclusion within one residential living community, the AZALEA Project (Arizona Alliance for LGBTQ2S+ Equity in Aging) continues to evolve as a broader statewide LGBTQ2S+ aging equity alliance. We will share emerging successes and lessons learned from an inaugural LGBTQ2S+ Aging Equity Forum and how these are informing future research development and design.

